# Bronchial angiogenesis in horses with severe asthma and its response to corticosteroids

**DOI:** 10.1111/jvim.16159

**Published:** 2021-05-28

**Authors:** Esther M. Millares‐Ramirez, Jean‐Pierre Lavoie

**Affiliations:** ^1^ Department of Clinical Sciences, Faculty of Veterinary Medicine University of Montreal Saint‐Hyacinthe Quebec Canada

**Keywords:** angiogenesis, asthma, collagen IV, horses, immunohistochemistry

## Abstract

**Background:**

Severe asthma in horses is characterized by structural changes that thicken the lower airway wall, a change that is only partially reversible by current treatments. Increased vascularization contributes to the thickening of the bronchial wall in humans with asthma and is considered a potential new therapeutic target.

**Objective:**

To determine the presence of angiogenesis in the bronchi of severely asthmatic horses, and if present, to evaluate its reversibility by treatment with corticosteroids.

**Animals:**

Study 1: Bronchial samples from asthmatic horses in exacerbation (7), in remission (7), and aged‐matched healthy horses. Study 2: Endobronchial biopsy samples from asthmatic horses in exacerbation (6) and healthy horses (6) before and after treatment with dexamethasone.

**Methods:**

Blinded, randomized controlled study. Immunohistochemistry was performed using collagen IV as a marker for vascular basement membranes. Number of vessels, vascular area, and mean vessel size in the bronchial lamina propria were measured by histomorphometry. Reversibility of vascular changes in Study 2 was assessed after 2 weeks of treatment with dexamethasone.

**Results:**

The number of vessels and vascular area were increased in the airway walls of asthmatic horses in exacerbation (*P* = .01 and *P* = .02, respectively) and in remission (*P* = .02 and *P* = .04, respectively) when compared to controls. In Study 2, the differences observed between groups disappeared after 2 weeks of treatment with corticosteroids because of the increased number of vessels in healthy horses.

**Conclusions and Clinical Importance:**

Angiogenesis contributes to thickening of the airway wall in asthmatic horses and was not reversed by a 2‐week treatment with corticosteroids.

**Abbreviations**
CS: corticosteroids
ECM: extracellular matrix
E_L_
: pulmonary elastance
R_L_
: pulmonary resistance

## 1 INTRODUCTION

Asthma in horses is an umbrella term encompassing various chronic inflammatory processes affecting the lower airways.^1^, ^2^ This syndrome is considered one of the most common conditions affecting adult horses.^3^ Severe asthma in horses is associated with excessive mucus accumulation, airway hyperresponsiveness, and structural changes of the airway wall (remodeling) leading to airflow obstruction and poor performance.^4^ This remodeling develops in response to chronic inflammation and includes epithelial hyperplasia, collagen deposition, and smooth muscle hypertrophy and hyperplasia.^5^, ^6^ These structural changes contribute to the impairment of lung function observed in asthmatic horses.^7^, ^8^, ^9^ In asthmatic human patients, an association between chronic inflammation of the airways and the development of angiogenesis, a process defined as the formation of new vessels from preexisting ones, has been identified.^10^, ^11^ Moreover, a study reported increased airway mucosal blood flow in the airways of asthmatic humans when compared to normal subjects.^12^ Similarities between asthma in humans and horses indicate that angiogenesis also may be present in affected horses.^5^ Importantly, airway remodeling in severe asthma of horses is only partially reversible with current treatments (eg, inhaled and systemic corticosteroids, bronchodilators).^13^, ^14^

The stimulus leading to angiogenesis in asthmatic humans is unclear.^15^ However, angiogenesis and chronic inflammation have been shown to be codependent.^16^ Furthermore, hypoxic pulmonary tissues release angiogenic growth factors such as vascular endothelial growth factor (VEGF) that contribute to the migration of endothelial cells from preexisting vessels to the surrounding extracellular matrix (ECM) to form solid sprouts. Inflammatory cells such as macrophages, mast cells, and fibroblasts also can lead to an increase in vessel growth,^17^ which may contribute to thickening of the bronchial wall. An in vitro study suggested that corticosteroids (CS) can reverse angiogenesis in horses, because they decrease the release of VEGF from equine pulmonary artery endothelial cells.^18^ Although several studies have identified the presence of angiogenesis in humans with asthma,^19^, ^20^, ^21^, ^22^ with an increased number of subepithelial vessels and vascular area in lung tissue,^11^, ^23^ literature on lung angiogenesis in asthmatic horses is scarce. A 2012 study^24^ showed an increased vascular density of superficial tracheal vessels in asthmatic horses, when evaluated by narrow‐band imaging. However, no differences were observed at the level of the bronchi, conflicting with results obtained in humans. The authors attributed those findings to a lack of sensitivity of the imaging technique to assess vascular changes.

Because inflammation and hypoxemia are common findings in horses with severe asthma, our objectives were to evaluate the presence of bronchial angiogenesis using immunohistochemistry to quantitatively determine the presence of mature bronchial vessels in the airways of asthmatic and age‐matched healthy horses, and to evaluate the angiogenic response to systemically administered CS. We hypothesized that an increased number of vessels would be observed in the airways of asthmatic horses when compared to controls, and that this process would be reversible by treatment with CS.

## 2 MATERIAL AND METHODS

### 2.1 Study design

Blinded, randomized controlled study. Study 1 evaluated the presence of angiogenesis in the central airways of severely asthmatic horses, and Study 2 examined its reversibility after a 2‐week treatment with CS.

### 2.2 Animals

#### 2.2.1 Study 1

Frozen bronchi from 14 horses previously diagnosed with severe asthma (research herd) and 7 healthy horses (teaching herd) from the Equine Respiratory Tissue Biobank (ERTB, www.ertb.ca, Faculty of Veterinary Medicine, University of Montreal) were studied. Information regarding the asthmatic status of the horses (clinical signs and standard pulmonary function), housing characteristics, and feeding regimen was available for review. Horses were divided into 3 groups based on history (respiratory signs such as increased respiratory effort at rest), bronchoalveolar lavage (BAL) fluid cytology, and antemortem lung function (pulmonary resistance [R_L_] and pulmonary elastance [E_L_]^25^, ^26^), as exacerbation (n = 7, R_L_ > 1 cmH_2_O/L/s, E_L_ > 1 cmH_2_O/L, BAL fluid neutrophils >25%), remission (n = 7, R_L_ ≤ 1 cmH_2_O/L/s, E_L_ ≤ 1 cmH_2_O/L, BAL fluid neutrophils <5%), and control (n = 7, no respiratory signs, R_L_ ≤ 1 cmH_2_O/L/s, E_L_ ≤ 1 cmH_2_O/L, BAL neutrophils <5%).^27^ All horses were stabled in a barn in individual stalls with wood shavings, except for 4 horses in remission that were at pasture. Stabled horses were fed hay twice daily whereas those on pasture were not supplemented hay with the exception of 1 horse that also received hay pellets. Turnout was allowed daily for all of the stabled horses. Horses were maintained in remission status for a minimum of 3 months either by decreasing dust exposure by turning them out into pasture (n = 4), or by the administration of inhaled CS (n = 3, fluticasone 2500 μg/q12h for 3‐4 months) before being sampled.

#### 2.2.2 Study 2

The experimental protocol and characteristics of the horses studied have been reported previously.^28^ In brief, subsegmental endobronchial biopsy samples were obtained from horses with severe asthma in exacerbation of the disease (n = 6) diagnosed based on standard lung function parameters and BAL fluid cytology results and from age‐matched controls (n = 6). Horses were stabled in the same barn and fed normal hay for 1 month to induce airway obstruction in asthma‐susceptible horses. Horses from both groups in Study 2 (healthy and asthmatic animals in exacerbation) then were treated with dexamethasone (Dominion Veterinary Laboratories Ltd, Manitoba, Canada) at a dosage of 0.06 mg/kg PO q24h for 2 weeks while being kept in the same environment.

Experimental procedures were performed in accordance with the Canadian Council for Animal Care and the research protocol was approved by the University of Montreal Animal Care Committee (Rech‐1578, Rech‐1716).

### 2.3 Immunohistochemistry

**FIGURE 1:**
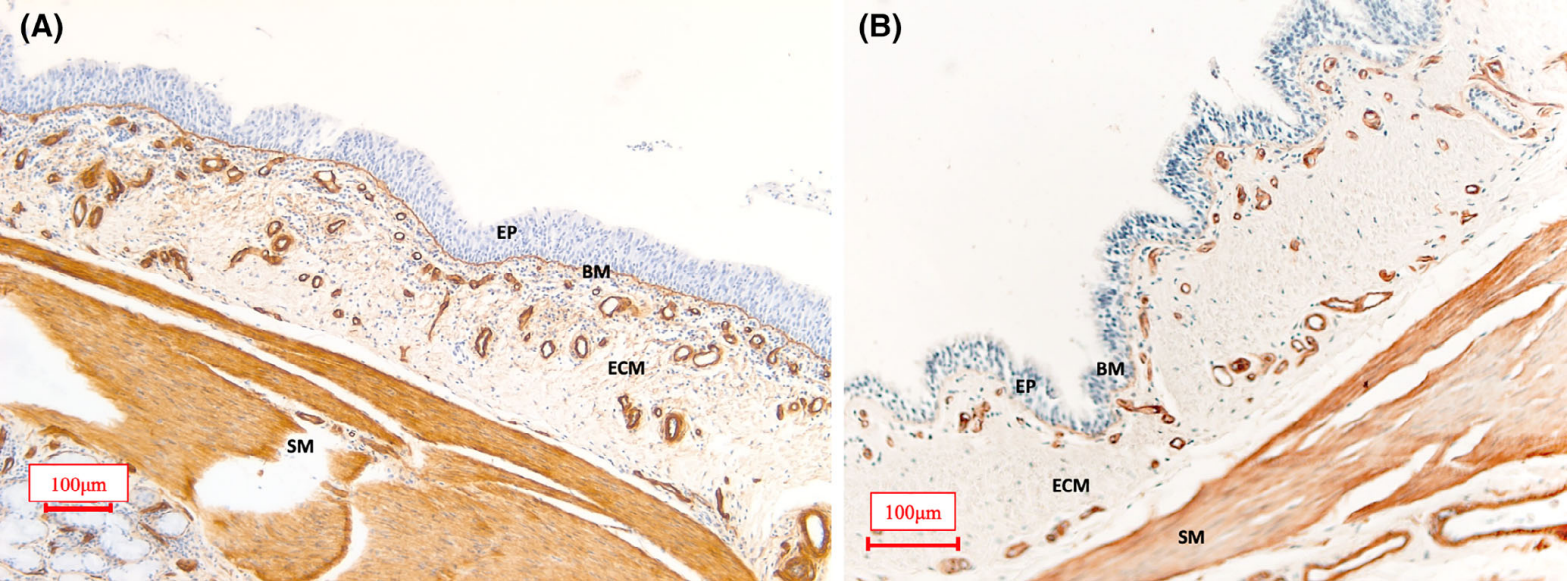
Representative image of a bronchus from an asthmatic horse (A) and a control horse (B) stained with collagen IV highlighting in brown the basement membrane of blood vessels in the airway lamina propria. The smooth muscle cells are also diffusely stained by collagen IV. BM, basement membrane; ECM, extracellular matrix; EP, epithelium; SM, smooth muscle. Scale bar 100 μm

Collagen IV, a major basement membrane protein, was used to outline the endothelial basement membrane.^10^ Two complete airways (Study 1) and 2 endobronchial biopsy samples (EBB‐Study 2) were studied for each horse. Equine bronchi specimens initially snap‐frozen in liquid nitrogen and maintained at −80°C were thawed in phosphate‐buffered saline (PBS 1×) at room temperature and cut into 0.5 cm samples. Tissue samples then were fixed for 24 hours in 4% formaldehyde and embedded in paraffin blocks until analyzed. All of the blocks were stored at room temperature before use. Histologic sections of 5 μm thickness were used for immunohistochemistry. Enzymatic antigen retrieval was performed using pepsin (Ready‐to‐use, ImmunoBioScience #AR‐6543‐0, Mukilteo, WA, USA). Collagen IV (Dilution 1 : 50, Mouse Anti‐Human, IgG1 monoclonal, Dako #M0785, Agilent Technologies, Mississauga, ON, Canada) was used as the primary antibody with an overnight incubation at 4°C. Donkey antimouse biotinylated (Dilution 1 : 1000, Jackson Immunoresearch Laboratory #715‐065‐150, West Grove, PA, USA) was used as a secondary antibody, and DAB peroxidase (Substrate kit, Vector Laboratories, Inc, #SK4100, Burlingame, CA, USA) was applied to produce a brown coloration of the bronchial ECM capillary basement membranes (Figure 1A,B). An isotype control antibody (Dilution 1 : 50, Mouse IgG, Sigma Aldrich, #M5409, Buffalo Grove, IL, USA) was used to confirm the specificity of the staining. Histologic slides were counterstained with Harry's hematoxylin and mounted using Leica Micromount (Surgipath Micromount Mounting Medium, Leica #3801731, Buffalo Grove, IL, USA).

### 2.4 Histomorphometric analysis

**FIGURE 2:**
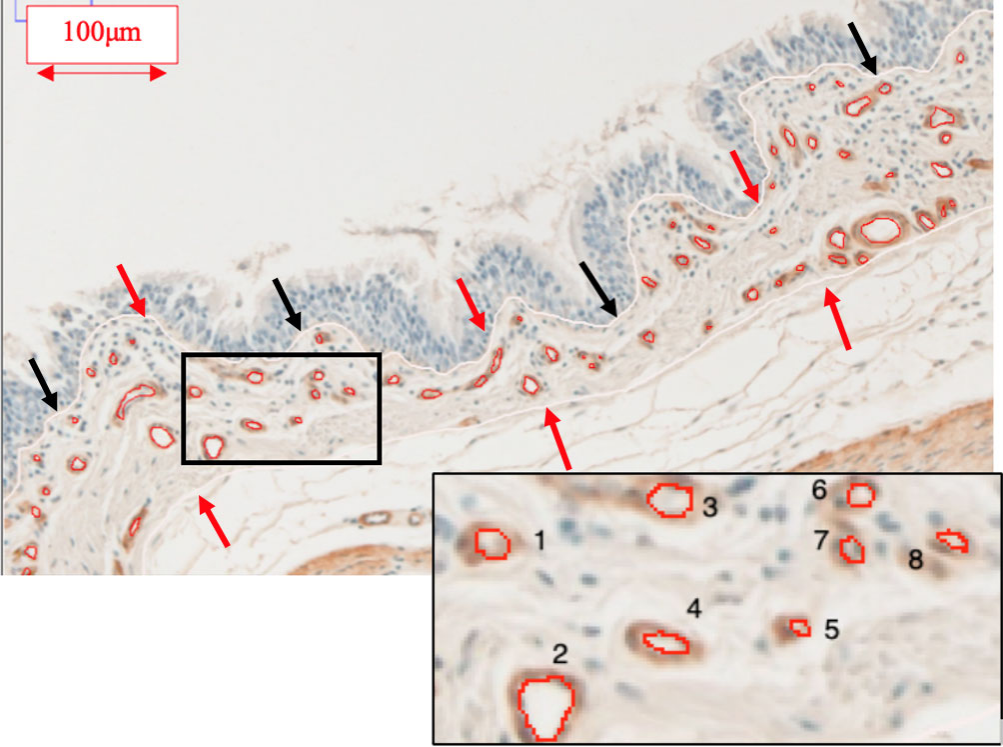
Example of the tracings for the calculation of the vascular area and the number of vessels in the airway lamina propria. A white line was firstly used to calculate basement membrane length (black arrows), and secondly, it was used to outline the extracellular matrix area (red arrows). Luminal circumference of each vessel included in a region of interest is outlined in red (vascular area). Each vessel was manually assigned a number for vessel count. Scale bar 100 μm

Histologic sections were evaluated on a 20× magnification and digitized using an automated upright microscope system with a FLIR camera (Life Science Research, Leica DM4000‐B, Buffalo Grove, IL, USA); (FLIR Integrated Imaging Solutions, Inc, Richmond, BC, Canada) and specialized software for image acquisition (Panoptiq 5.0, ViewsIQ, Inc, Vancouver, BC, Canada). The area between the epithelial basement membrane and a maximum distance of 150 μm in the lamina propria was analyzed in each region of interest (ROI), as previously described.^11^, ^23^ Six ROI with an area of 1 mm^2^ were randomly selected using number randomization software (https://www.random.org) to analyze blood vessels as described in previous quantitative studies.^23^, ^29^ A software analysis system (Image J, Version 1.52p, National Institutes of Health, Bethesda, Maryland) was used to evaluate the number of vessels, vascular density, vascular area, and mean vessel size for Study 1, and number of vessels and mean vessel size for the second phase of the study (Figure 2). Additionally, the epithelial basement membrane length of each section analyzed was measured manually by tracing using a freehand line tool to ensure that the images analyzed were of approximately similar size. The number of vessels in each ROI was manually counted, and the values obtained were corrected by the ROI's ECM surface area (Study 1) or by the corresponding epithelial basement membrane length (Study 2). The vascular surface area was computed directly by the software after manual tracing of the area internal to the vascular endothelial basement membrane of each vessel using the freehand selection tool. To determine the percentage of vascular density, the vascular area previously calculated was divided by the total ECM area and multiplied by 100. Finally, an estimation of mean vessel size was calculated by dividing the vascular area by the number of vessels. The values obtained for the 6 ROIs of each airway were averaged, resulting in a single value for each parameter and each horse. The operator (Esther Millares Ramirez) was blinded to group allocation of the samples during the analysis.

### 2.5 Statistical analysis

Heterogeneity among groups for age and weight was analyzed by 1‐way analysis of variance (ANOVA) and with the exact Chi‐squared test for sex. For the first study, a 1‐way ANOVA was used to examine differences among the 3 groups for R_L_ and E_L_ and histomorphometric traits. This analysis was followed by contrasts between pairs of means, adjusting the alpha level downward using the Benjamini‐Hochberg procedure. Values obtained in the second phase of the study were compared between 2 groups, control and exacerbation, using a 2‐sample *t* test. Values from the different groups (Study 2) at each time point were compared using a 2‐way repeated‐measures ANOVA. All statistical calculations were performed using computer software (GraphPad Prism 7.0, GraphPad Software, Inc, La Jolla, California). Statistical significance was set at *P* ≤ .05.

## 3 RESULTS

### 3.1 Study 1

#### 3.1.1 Animals

Sixteen mares and 5 geldings were studied, with a weight (mean ± SD) of 504 ± 74.5 kg and age of 16.8 ± 5.1 years. No association was observed between sex and group (*P* = .3). Likewise, mean age (*P* = .14) and mean weight (*P* = .35) did not differ significantly among diagnosis groups (exacerbation, remission, control; *P* > .05).

#### 3.1.2 Lung function

**FIGURE 3:**
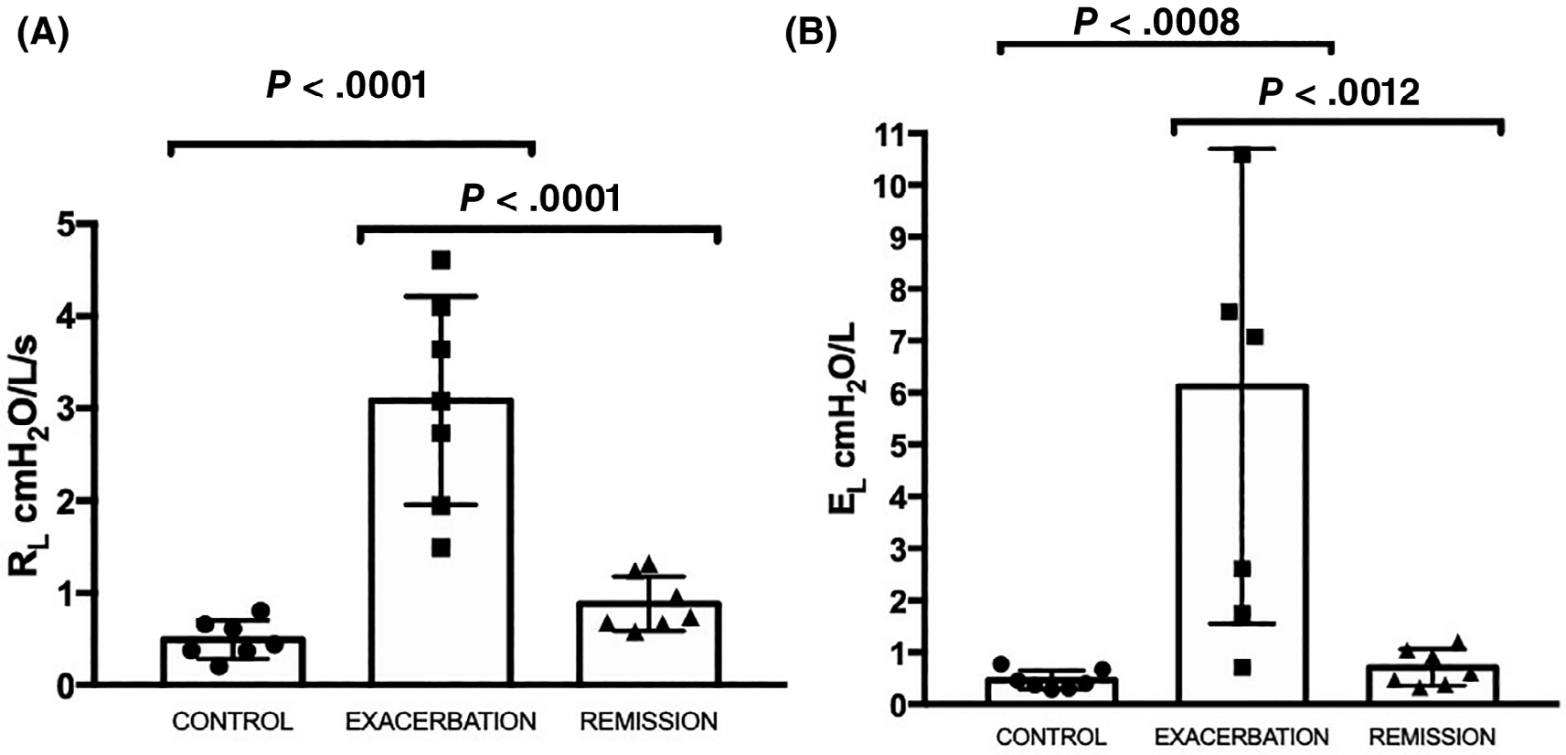
(A) Pulmonary resistance (R_L_) and (B) pulmonary elastance (E_L_) measured in control (n = 7) and asthmatic horses in exacerbation (n = 7) and remission (n = 7). The error bars indicate the standard deviation of the mean. Significantly higher R_L_ and E_L_ values were observed in horses in exacerbation when compared with control (*P* < .0001, *P* = .0008, respectively) and remission (*P* < .0001, *P* = .0012, respectively)

As expected because they were inclusion criteria, significantly higher R_L_ and E_L_ were observed in asthmatic horses in exacerbation when compared with controls (*P* < .01) and remission (*P* < .01; Figure 3A,B).

#### 3.1.3 Histomorphometric analysis

**FIGURE 4:**
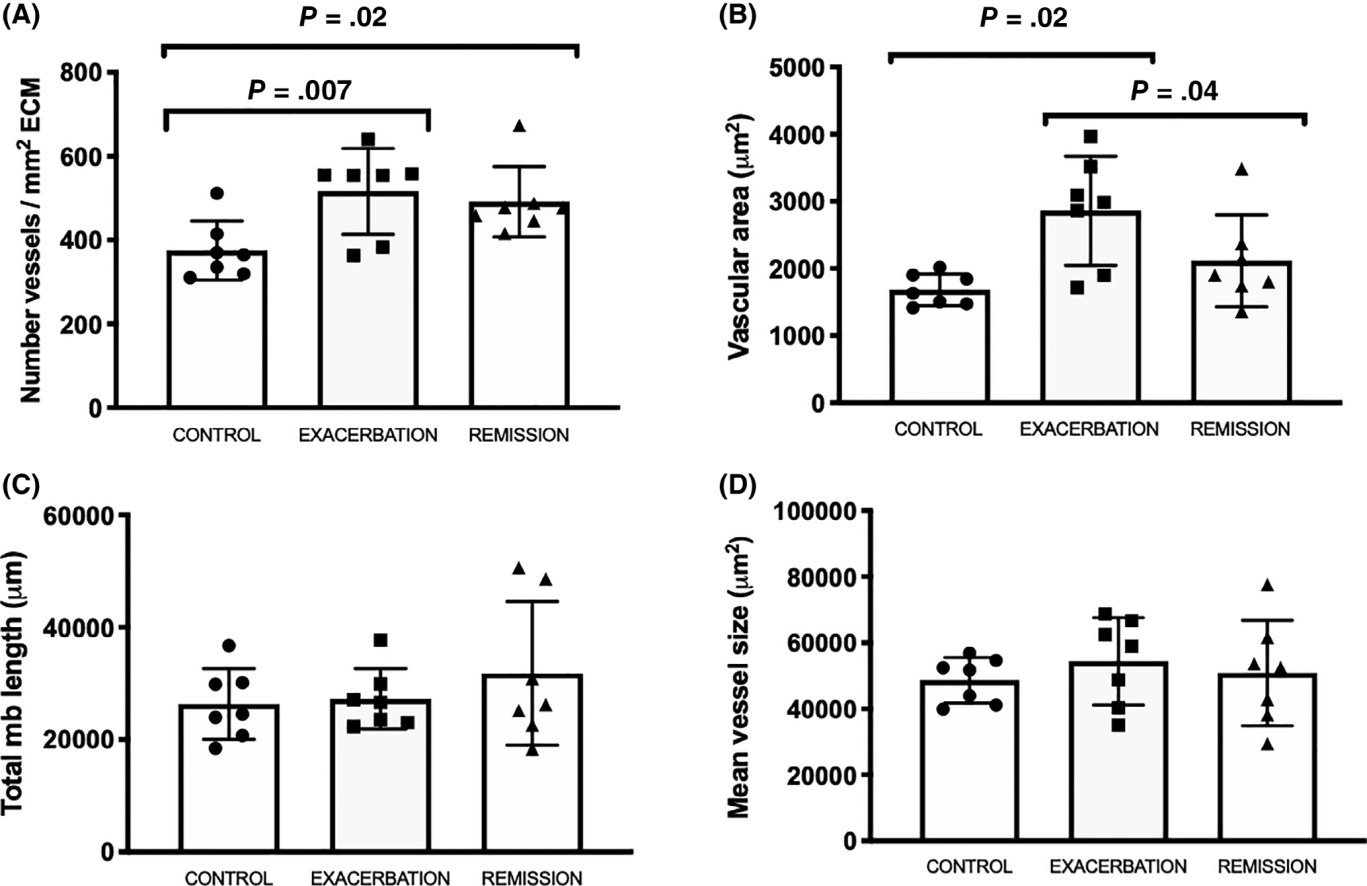
Histomorphometric results of Study 1. A, Total number of bronchial vessels per μm^2^ of extracellular matrix (ECM) in control and asthmatic horses in exacerbation and remission. An increased number of vessels in horses in exacerbation (*P* = .007) and remission (*P* = .02) when compared to control horses was observed. B, Vascular area of control and asthmatic horses in exacerbation and remission. An increased vascular area was observed in horses in exacerbation when compared to the control (*P* = .02) and remission horses (*P* = .04). C, Total epithelial basement membrane length values of control and asthmatic horses in exacerbation and remission. No differences were observed between the 3 groups. D, Mean bronchial vessel size of control and asthmatic horses in exacerbation and remission. No differences were observed between the 3 groups. The error bars indicate the standard deviation of the mean

The number of vessels corrected by the surface area of ECM was significantly increased in asthmatic horses in either exacerbation (*P* = .01; mean difference, −140 vessels/mm^2^) or remission (*P* = .02; mean difference, −120 vessels/mm^2^) when compared to control horses (Figure 4A). Furthermore, vascular area (measured by tracing the area internal to the vascular endothelial membrane of each vessel) was significantly increased in asthmatic horses in exacerbation when compared to controls (*P* = .02; mean difference, −1180 μm^2^) or to horses in remission (*P* = .04; mean difference, +747.3 μm^2^; Figure 4B). No difference was observed among groups in epithelial basement membrane length (*P* = .86; Figure 4C) or mean vessel size (vascular area divided by number of vessels; *P* = .4; Figure 4D).

### 3.2 Study 2

#### 3.2.1 Animals

Six mares and 6 geldings were evaluated, with a weight of 493.8 ± 51.6 kg and age of 14.3 ± 6.2 years. Additional details for Study 2 are reported elsewhere.^28^

#### 3.2.2 Lung function

As previously described,^28^ the significant differences in R_L_ observed at baseline between horses in exacerbation and healthy controls were lost after 2 weeks of treatment with dexamethasone.

#### 3.2.3 Histomorphometric analysis

**FIGURE 5:**
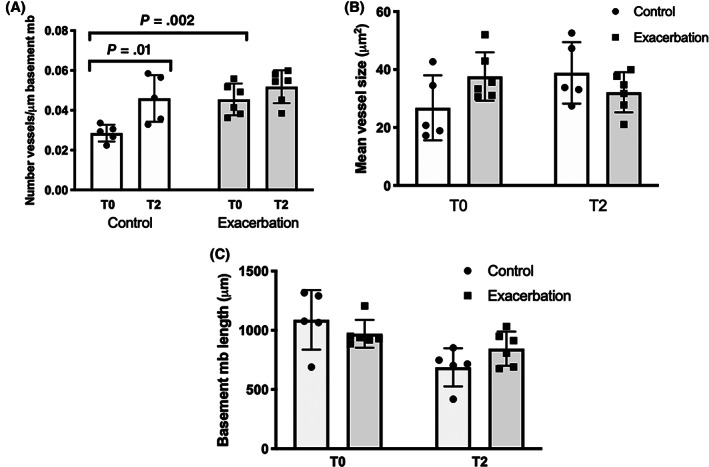
Histomorphometric results of Study 2. A, Total number of bronchial vessels per μm of epithelial basement membrane in control and asthmatic horses in exacerbation. A statistically significant increase in the number of vessels was observed in horses in exacerbation (*P* = .002) when compared to control horses at baseline. No significant differences were observed between control and exacerbation groups (*P* = .35) after 2 weeks of treatment because of an increase in the number of vessels in control horses (*P* = .01) after 2 weeks of treatment with dexamethasone. The number of vessels of horses in exacerbation remained unchanged. B, Mean bronchial vessel size of control and asthmatic horses in exacerbation. No significant differences were observed between groups at baseline (*P* = .1) or at week 2 (*P* = .24). C, Total epithelial basement membrane length values of control and asthmatic horses in exacerbation. No significant differences were observed between groups at baseline (*P* = .3) or at week 2 (*P* = .12). The error bars indicate the standard deviation of the mean

An increased number of vessels was observed in asthmatic horses in exacerbation (*P* = .002; 95% confidence interval [CI], 0.01 to 0.03) in comparison to control horses at baseline. This difference was lost after 2 weeks of treatment with CS (*P* = .35; 95% CI, −0.01 to 0.02; Figure 5A). This was the result of an increased number of vessels in the control horses after treatment with dexamethasone, as shown by intragroup differences (*P* = .01; 95% CI, −0.02 to −0.005). The number of vessels in the lamina propria in asthmatic horses was unaffected by CS. No difference was observed in mean vessels size or epithelial basement membrane length when comparing control and exacerbation horses at baseline (*P* = .1 and *P* = .3, respectively) or after 2 weeks of treatment with dexamethasone (*P* = .24 and *P* = .12, respectively; Figure 5B,C).

## 4 DISCUSSION

Angiogenesis is defined as the development of new vessels from preexisting ones. The codependence between angiogenesis and chronic inflammations plays a fundamental role in neoplasia and vascular malformations,^20^, ^21^ and in chronic lung diseases such as asthma.^22^, ^30^ The contribution of angiogenesis to the clinical signs in asthma has not been completely elucidated. Regardless of the underlying cause, a chronic inflammatory state stimulates proliferation and migration of inflammatory cells to the inflamed site, leading to organ damage and repair, potentially compromising oxygenation of affected tissues. The resulting hypoxia triggers the release of proangiogenic factors by inflammatory and structural cells.^19^ Angiogenesis in turn can contribute to maintaining or amplifying inflammation in diseases such as asthma by transporting cells, oxygen, and nutrients to the site of inflammation.^16^, ^31^

Our study provides the first evidence of angiogenesis occurring in bronchial tissues of the central airways in asthmatic horses. The increased number of vessels in asthmatic horses, either in exacerbation or remission, when compared to control horses, and the increased vascular area in horses in exacerbation are in agreement with observations in children and adults with mild to moderate asthma.^11^, ^23^, ^32^ Both groups of asthmatic horses had an increased number of vessels, but increased vascular area was observed only in horses in exacerbation when compared to controls. This absence of difference between the remission and control groups could be a consequence of these vessels being smaller in size although mean vessel size was not statistically different among the 3 groups or also could be related to the administration of inhaled CS for a minimum of 3 months before sample collection in 3 of the horses in the remission group. In addition, mean vessel size is considered to be an estimate, because correction for vessel dilatation was not performed because of the difficulty of measuring the diameter of the vessels in a large percentage of the vessels (because of folding and elongation of vessels), which could have contributed to the differences observed. Epithelial basement membrane length was used as a control variable to ensure that both groups had similar areas analyzed. This was confirmed by the absence of differences between groups in both Study 1 and Study 2. Because of the differences in morphology between the bronchial samples used in Study 1 (main bronchi) and Study 2 (endobronchial biopsy samples), the number of vessels in the first study was corrected by the area of ECM analyzed, whereas in Study 2 this variable was corrected by the epithelial basement membrane length.

Conventional treatment for asthma in horses and humans includes antigen avoidance and administration of CS and bronchodilators.^33^, ^34^, ^35^ However, these treatments only partially reverse the airway remodeling observed in asthmatic horses.^13^, ^36^ Contrary to what we had hypothesized, the number of vessels in asthmatic horses did not decrease after treatment. Furthermore, an increased number of vessels was observed in control horses after 2 weeks of treatment with dexamethasone. Studies in human asthmatics have shown decreased angiogenesis with CS.^37^, ^38^, ^39^ However, these apparent discrepancies may be a result of species differences. Indeed, a recent study comparing the responses of mice and horses to glucocorticoids in an in vitro aortic ring model of angiogenesis suggested an upregulation of proangiogenic pathways in the horses, whereas it was inhibited in mice.^40^ Vessel outgrowth was evident after 3 days and was maintained for 1 week. Furthermore, upregulation of proangiogenic growth factor VEGF signaling was observed in equine vessels exposed to cortisol.^40^ This finding might explain why in our study, healthy horses had an increase in the number of vessels with CS. Nevertheless, these results contrast with another study in which dexamethasone inhibited the expression of VEGF by equine pulmonary artery endothelial cells stimulated with interleukin‐4,^18^ a cytokine shown to be upregulated in horses with asthma,^41^ suggesting that the angiogenic response to CS also may be agonist dependent. Furthermore, the increased number of vessels after treatment was seen only in healthy horses rather than asthmatic horses. This could be a result of bronchial vessels already being augmented in comparison to baseline in horses with asthma, which may suggest that the capability of vessels to undergo angiogenesis might have reached a plateau or that angiogenesis is not decreased but only controlled by CS administration.

Immunohistochemistry allows identification and localization of molecules of interest by the immunolabeling of specific antigen‐antibody reactions.^42^, ^43^ Although manipulations and assessment of results can be challenging using this technique,^44^, ^45^ it is considered the gold standard method when quantifying bronchial vasculature.^10^ In our study, the basement membrane protein, collagen IV, was used as a biomarker for evaluating the presence of angiogenesis.^10^, ^11^ This antibody highlights the vessel by staining the basement membrane supporting the endothelium.^11^ This marker is considered superior to endothelial biomarkers such as platelet endothelial cell adhesion molecule‐1 (PECAM‐1) and factor VII,^20^, ^47^ because it is considered selective for the vascular basement membrane. Lymphatic vessels also might be positively stained depending on the epitopes of collagen IV targeted by the antibody. However, the staining likely would be weak in lymphatics because of their incomplete basement membrane and absence of pericytes.^46^ Furthermore, the antibody used in our study was reported to be specific for blood vessels in humans.^47^

A limitation of our study was the absence of milder clinical cases of asthma, and therefore results may not be directly applicable to horses with milder disease. Furthermore, our study was performed on a small number of animals. The strict experimentally controlled environmental conditions under which horses with severe asthma and control horses were maintained during the study necessitated limiting the number of animals required to reach statistical differences. Nevertheless, the study may not have had the power to detect smaller changes in some of the variables that were evaluated.

In conclusion, our study determined that in addition to pulmonary structural changes, an increase in vascularity occurs in horses with severe asthma. These results may offer a new opportunity for treatment by targeting angiogenesis and possibly decreasing inflammation and remodeling of the asthmatic airways. Furthermore, treatment with CS increased angiogenesis in healthy horses, whereas angiogenesis was maintained in asthmatic horses suggesting that conventional treatment does not decrease the number of bronchial vessels observed in the ECM. Lastly, the proangiogenic effects of CS observed in our study may have implications for diseases of horses associated with increased cortisol secretion or when CS are administered chronically.

## CONFLICT OF INTEREST DECLARATION

Authors declare no conflict of interest.

## OFF‐LABEL ANTIMICROBIAL DECLARATION

Authors declare no off‐label use of antimicrobials.

## INSTITUTIONAL ANIMAL CARE AND USE COMMITTEE (IACUC) OR OTHER APPROVAL DECLARATION

All experimental procedures were performed in accordance with the Canadian Council for Animal Care and the research protocol was approved by the University of Montreal Animal Care Committee (Rech‐1578).

## HUMAN ETHICS APPROVAL DECLARATION

Authors declare human ethics approval was not needed for this study.
